# HR-ACT (Human–Robot Action) Database: Communicative and noncommunicative action videos featuring a human and a humanoid robot

**DOI:** 10.3758/s13428-025-02910-0

**Published:** 2026-01-12

**Authors:** Tuǧçe Nur Pekçetin, Gaye Aşkın, Şeyda Evsen, Tuvana Dilan Karaduman, Badel Barinal, Jana Tunç, Cengiz Acarturk, Burcu A. Urgen

**Affiliations:** 1 Department of Cognitive Science, Graduate School of Informatics, Middle East Technical University, Çankaya/Ankara, Türkiye; 2 Department of Psychology, Bilkent University, Çankaya/Ankara, Türkiye; 3 Department of Neuroscience, Bilkent University, Çankaya/Ankara, Türkiye; 4 Department of Psychology, Middle East Technical University, Çankaya/Ankara, Türkiye; 5 Center for Cognitive Science, Jagiellonian University, Kraków, Poland; 6 Aysel Sabuncu Brain Research Center and National Magnetic Resonance Research Center (UMRAM), Bilkent University, Çankaya/Ankara, Türkiye

**Keywords:** Action perception, Social robotics, Normative data, Human–robot interaction, Communicative actions

## Abstract

We present the HR-ACT (Human–Robot Action) Database, a comprehensive collection of 80 standardized videos featuring matched communicative and noncommunicative actions performed by both a humanoid robot (Pepper) and a human actor. We describe the creation of 40 action exemplars per agent, with actions executed in a similar manner, timing, and number of repetitions. The database includes detailed normative data collected from 438 participants, providing metrics on action identification, confidence ratings, communicativeness ratings, meaning clusters, and *H* values (an entropy-based measure reflecting response homogeneity). We provide researchers with controlled yet naturalistic stimuli in multiple formats: videos, image frames, and raw animation files (.qanim). These materials support diverse research applications in human–robot interaction, cognitive psychology, and neuroscience. The database enables systematic investigation of action perception across human and robotic agents, while the inclusion of raw animation files allows researchers using Pepper robots to implement these actions for real-time experiments. The full set of stimuli, along with comprehensive normative data and documentation, is publicly available at https://osf.io/8vsxq/.

## Introduction

For many species, survival depends on their ability to observe their surroundings and adapt their behaviors accordingly. A critical component of this observational process is the ability to interpret others’ actions. This skill is not only crucial for survival, but it also forms the basis of social cognition, particularly in primates. In humans, action perception is closely associated with higher-order social skills such as communication, intention understanding, and empathy, which are vital for complex interactions and social coordination (Blake & Shiffrar, 2007).

As our social environment evolves to include artificial agents alongside humans and animals, understanding how humans perceive and interpret robot actions has become a critical research focus (Cross et al., 2019). This line of investigation spans domains of cognitive science and neuroscience, exploring how humans process the behaviors of artificial agents at various levels, ranging from basic motion perception to complex social understanding (Henschel et al., 2020). Such studies have implications for both the theoretical understanding of human cognition and the practical development of social robots. Research has revealed that while humans process robot actions using mechanisms similar to those used for human actions, they also show distinct cognitive and neural response patterns unique to artificial agent observation (Cross & Ramsey, 2021).

Recent studies examining how robot-related factors influence action perception indicate that multiple elements affect how humans process and respond to robot behaviors. Besides factors such as perceived similarity (Waytz et al., 2010) and appearance or the degree of anthropomorphism (Gray & Wegner, 2012), robots’ behaviors and capabilities significantly influence how humans perceive and interact with them. For instance, robots were reported to elicit stronger responses when they exhibit a range of socially interactive behaviors (Fraune et al., 2020), such as emotional expression (Złotowski et al., 2014), or gestures (Salem et al., 2011). Furthermore, the specific type of action performed by a robot has been reported to have varying effects on how they are perceived (Pekçetin et al., 2024a; Saltik et al., 2021; Złotowski et al., 2014). However, there are also studies indicating that the nature of a robot’s behavior does not alter these perceptions (Straub, 2016). These conflicting findings highlight the complexity of how robot actions influence human perception and response patterns during human–robot interaction (HRI). The selection of diverse stimuli and the use of various experimental designs may also contribute to these discrepancies.

Even for human actions, not all actions are perceived identically. The influence of action type has also become relevant in neuroscientific investigations, particularly following recent studies that emphasize the significance of testing actions from different categories (Abdollahi et al., 2013; Ferri et al., 2015; Urgen & Orban, 2021). Actions are categorized into distinct classes based on shared sensorimotor transformations or motor goals (Orban et al., 2021). For instance, the manipulative action class includes behaviors such as *squeezing* or *dragging an object*, aimed at altering the object’s form or position. Conversely, the interpersonal action class involves actions like *chasing* or *showing aggression*, intended to influence others’ behavior (Orban et al., 2021). Recent work reveals that different classes of actions, such as manipulative or communicative, produce distinct neural representations, particularly in the parietal cortex (Urgen & Orban, 2021).

In cognitive neuroscience literature, various HRI studies utilized different types of robotic stimuli, though many have significant limitations. For instance, an early study by Tai et al. (2004) used simple robotic movements, specifically a robotic arm and a human arm performing *reach-and-grasp* actions. Similarly, Gazzola et al. (2007) compared neural responses to human, anthropomorphic, and mechanical robot hands executing goal-directed *grasping* movements. A more sophisticated approach emerged with the widely used Saygin-Ishiguro database, which provided matched videos of a human, an android, and a mechanical robot performing identical everyday actions (Saygin et al., 2012; Urgen et al., 2013, 2018; Urgen & Saygin, 2020). These actions included tasks such as *waving*, *drinking from a cup*, and *wiping a surface*. More recently, Di Cesare et al. (2020) examined brain activity using videos of a human and the iCub robot performing a *passing* action with different kinematic profiles intended to convey *gentle* or *rude* intentions. However, while valuable, these studies have largely relied on a limited repertoire of relatively simple actions, representing only a narrow range of action classes.

Consequently, the perception of a robot might vary depending on whether it is observed performing manipulative actions like *dragging an object* or *wiping a surface*, or when it is engaged in communicative actions requiring social interactions like *hand-waving* or *chasing someone*. Recent reviews highlight the need for complementary research approaches to understand these perceptual differences (Cross & Ramsey, 2021; Henschel et al., 2020). Controlled cognitive neuroscience studies provide precise measurements of how humans process robot actions, and research in naturalistic settings reveals how these perceptions influence real-world human–robot interactions (Thellman et al., 2022).

**Fig. 1:**
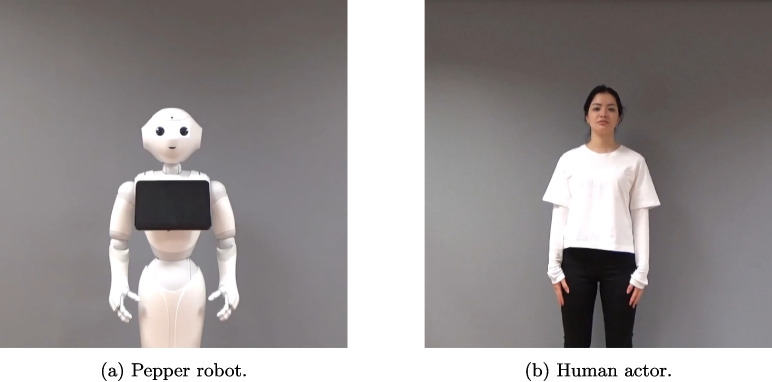
The two actors in the HR-ACT Database, photographed against a dark gray background to ensure comparable video presentations

The systematic investigation of robot action perception requires carefully selected stimuli. While several action databases exist, they predominantly focus on human actions, as detailed in a comprehensive review by David et al. (2023). These databases are typically designed for computer vision applications, focusing on algorithm training and testing rather than standardized experimental stimuli. Across these resources, typical content spans everyday object-directed actions (e.g., *wiping a surface*, *pouring*, or *drinking*), gesture-based communicative acts (e.g., *waving*), and interaction sequences (e.g., *handovers/passing*). Recent efforts to create standardized action stimuli include a large video set of 100 human actions validated with functional Magnetic Resonance Imaging (Urgen et al., 2022), and specialized databases featuring communicative (Manera et al., 2010) and noncommunicative (Zaini et al., 2013) actions in point-light display format. While the field of HRI benefits from resources such as the Anthropomorphic roBOT (ABOT) Database (Phillips et al., 2018), which provides a comprehensive collection of standardized images of anthropomorphic robots, it does not feature dynamic action videos. Recently, a large-scale robotics dataset Fourier ActionNet (Fourier ActionNet Team, 2025), which offers extensive videos of humanoids performing complex tabletop manipulative actions (e.g., *pick-and-place*), was introduced. While this dataset provides a valuable resource by providing a rich source of training data for developing and benchmarking robot policies, it was not specifically designed or validated as a stimulus set for human perception experiments. Consequently, there remains a notable absence of stimulus sets featuring dynamic robot actions tailored for HRI research. This gap is particularly significant as existing human action or point-light display databases cannot be readily adapted for robot-focused research. To address this limitation, we have developed a new database incorporating standardized communicative and noncommunicative action stimuli performed by both a robot and a human actor.

In this study, we present the HR-ACT (Human–Robot Action) Database, comprising 80 videos featuring a human and a humanoid robot performing communicative and noncommunicative actions. Following established frameworks in action research (Ekman & Friesen, 1969; McNeill, 1985), we classify actions based on their social intent and recipient orientation. *Communicative* actions are nonverbal behaviors intended to convey meaning to a recipient within a social interaction context (Manera et al., 2010). *Noncommunicative* actions, in contrast, are object-oriented or environmental responses that serve functional purposes (Zaini et al., 2013). While more granular action classifications exist for human actions, we adopted this broader communicative/noncommunicative distinction to align with frequently used categorizations in the HRI literature and to account for the current limitations in robot mobility and affordances.

The HR-ACT Database makes several unique contributions to the field. First, it addresses the need for a comprehensive action database that includes both human and robot performances of the same actions, enabling direct comparisons. Second, it supports the field’s growing demand for naturalistic stimuli while maintaining experimental control. Third, by featuring the raw animation files (.qanim format) alongside the action videos and frames, it enables researchers to implement these actions on the Pepper robot for real-time interaction studies. The database’s design, incorporating both communicative and noncommunicative actions performed by both human and robot agents, makes it particularly valuable for investigating questions ranging from basic action perception to complex social cognition in human–robot interaction. The following sections detail our methodological approach, including action selection criteria, stimulus creation procedures, normative data collection, and validation results.

## Method

### Stimulus creation

We developed a database of communicative and noncommunicative actions by systematically selecting both the agents and the actions. The initial phase involved selecting appropriate actors and identifying actions that could be feasibly executed by both agents, taking into account their physical capabilities and limitations.

#### Selection of agents

To enable direct comparisons in future studies, we designed the database to include matched action videos from both a robot and a human actor. This required careful selection of actors with comparable physical properties, particularly in terms of body size and posture, who could effectively perform the range of intended actions. Figure 1 illustrates both actors as they appeared in the study videos.

##### Pepper robot

Pepper was introduced as the world’s first social humanoid robot, which can recognize faces and basic human emotions (Aldebaran, 2025). Pepper is equipped with a tablet computer on its torso, which displays content to support its speech capabilities and highlight messages (see Fig. 1a

There are several properties of the Pepper robot that made it an ideal candidate for our research. First, Pepper is designed as a gender-neutral humanoid robot, featuring a face with two eyes, a mouth, a nose-like structure, and two ear-like speakers, along with a torso and two arms with hands, making its form comparable to human body proportions. Second, Pepper’s head and arm joints provide specific ranges of motion for natural interaction. The head’s yaw and pitch capabilities enable looking around and nodding, while the arm and wrist joints allow for both communicative and noncommunicative actions. Third, currently, there are few alternative robots that match Pepper’s combination of height (120 cm), movement capacities, and anthropomorphic features. Lastly, and crucially for our study, the robot’s open and fully programmable API via the QiSDK (Aldebaran, 2017) enables programming through Android Studio (Google, 2021), with the Pepper SDK Plugin (SoftBank Robotics, 2020b) providing sample animation files and an animation editing interface for creating or modifying movements.

##### Human actor

The human actor, a female graduate student from the Cognitive Computational Neuroscience Lab (CCN Lab) at Bilkent University, was selected based on three key criteria. First, her previous acting experience and training provided the necessary performance skills. Second, as a lab member, she was familiar with the study objectives and the Pepper robot’s capabilities. Third, her physical stature required only minor camera angle adjustments to match the robot’s proportions in recordings (see Fig. 1b). The actor provided informed consent for the publication of her images and videos, and was compensated for her participation.

#### Action selection and filtering

The final set of 40 actions for the HR-ACT Database was determined through a multi-stage filtering process designed to ensure theoretical breadth, feasibility, and recognizability. Our process began by generating a broad pool of candidate actions drawn from three key sources. First, we drew inspiration from the validated stimulus sets of Manera et al. (2010), which provided 20 communicative actions (e.g., *‘stop it’*, *‘I am angry’*) and Zaini et al. (2013), which provided a list of 43 communicative and noncommunicative pantomimed actions (e.g., *clapping*, *dancing*). Although these databases used point-light displays of human actions, their detailed action descriptions provided valuable guidance for adapting movements for our context and purposes. Second, to broaden our possible action pool and leverage the robot’s existing capabilities, we identified potentially suitable pre-existing animations from the Pepper SDK’s QiSDK tutorials repository (Aldebaran, 2017). Finally, to ensure theoretical robustness, this initial pool was conceptually guided by the action classes proposed by Orban et al. (2021), ensuring coverage across categories such as *Manipulation*, *Locomotion*, and *Interpersonal* actions.

This rich list of candidate actions was then screened for feasibility against the Pepper robot’s physical constraints. Most notably, despite having sensors on its finger joints, Pepper’s hands function only as single units without independent finger movement capabilities. This restriction eliminated actions which require precise finger control, such as a *threatening* gesture or a *zipping* motion. It particularly affected our ability to implement common communicative gestures that rely on specific finger positions (e.g., *‘one moment,’* *‘over there,’* *‘good job,’* *‘I am watching you’*). Furthermore, the absence of a wrist pitch axis restricted certain communicative actions, such as the *‘stop’* gesture, while the centrally mounted tablet on the robot’s torso prevented arm-crossing movements like *hugging* or the *‘it is cold’* gesture. The unified lower body design, lacking separate legs, required us to simulate locomotion through upper body movements for actions such as *jogging*, *swimming*, and *dancing*. Although Pepper features expressive elements like blinking and color-changing eyes, its limited facial expression capabilities led us to rely primarily on body movements, resulting in predominantly pantomime-like actions. This feasibility screening, which involved carefully weighing each candidate action against these physical limitations, narrowed our initial pool to a viable set of 50 actions for development.

**Fig. 2:**
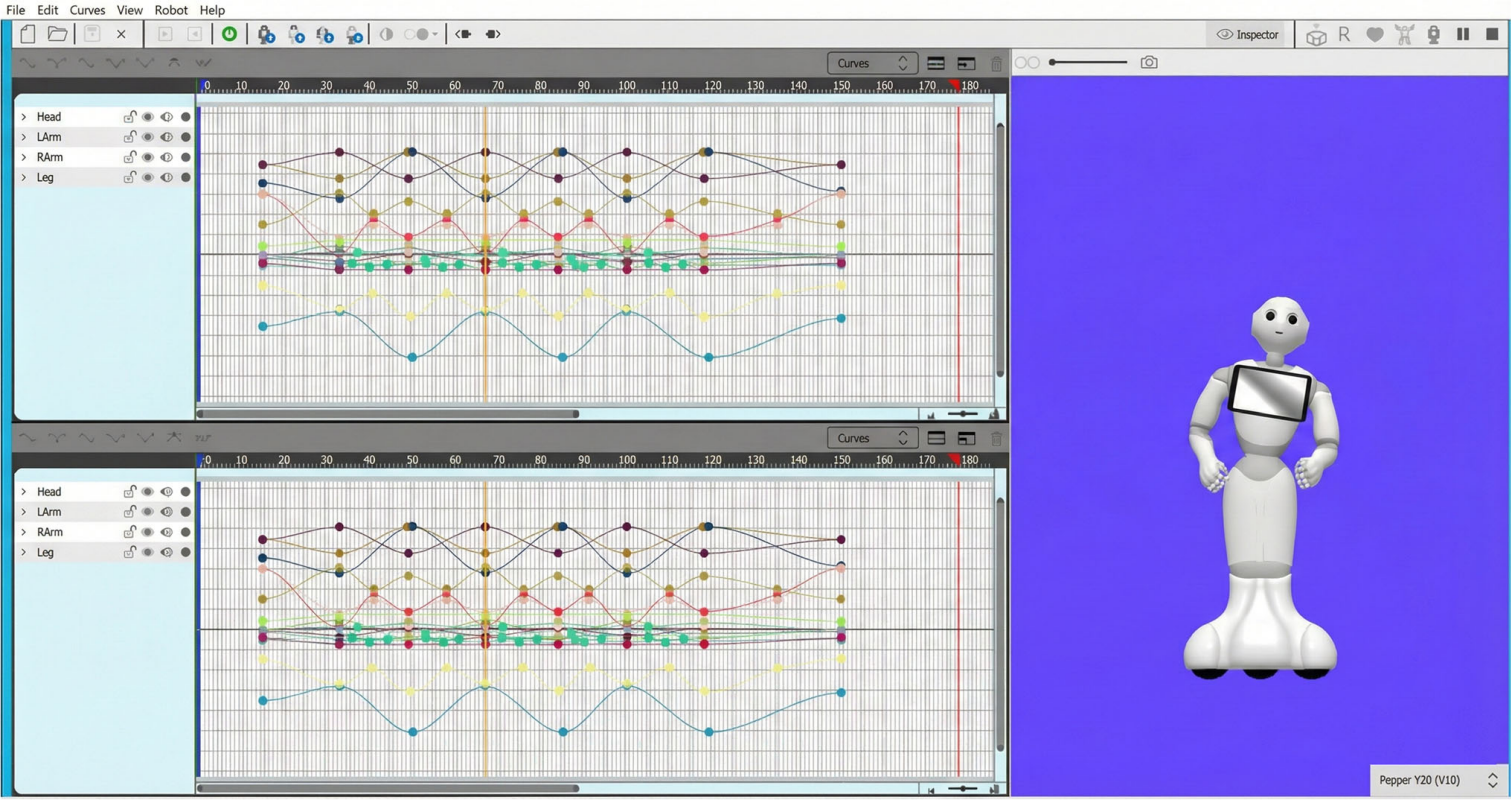
The Animation Editor interface for the *dancing* action in the HR-ACT Database. The *left panel* shows keyframe controls for manipulating the robot’s head, arms, hands, and lower body movements across roll, pitch, and yaw axes. The *right panel* displays the emulator robot for previewing animations before physical implementation

We then developed the robot animations using the Animation Editor in Pepper SDK (SoftBank Robotics, 2020a), either creating new animations from scratch or modifying existing ones from the repository. The process involved manipulating keyframes to achieve our intended actions, as illustrated for the *dancing* action in Fig. 2. Despite the emulator’s utility, physical testing on the actual robot proved essential, as certain limitations, such as abnormal movement speeds or physically impossible actions, became apparent only during real-world implementation.

Following the animation creation process, these 50 developed animations were subjected to a two-stage filtering process to finalize the action set. The first stage was a rigorous internal review by our research team to assess recognizability. This internal review revealed that eight of the pre-existing animations from the Pepper SDK were consistently ambiguous or poorly understood. The excluded actions were: *comb hair*, *bump*, *smelly*, *rub belly*, *nauseation*, *happy*, *sad*, and *gorilla* (an action depicting hitting the torso). This left a refined set of 42 actions.

In the second stage, these 42 actions underwent a formal pre-norming study. We conducted face-to-face sessions with four external reviewers representing different expertise levels: two experts (a 3D designer with an art background and a PhD candidate in human–robot interaction) and two novices (a psychology master’s student and an undergraduate psychology student). Each reviewer assessed 21 animations in separate sessions, providing detailed feedback on action identification, communicative intent, and execution quality (rated 1–5). The expert reviewers focused on technical aspects such as motion naturalness, while novice reviewers evaluated comprehensibility. Based on this feedback, we excluded two final actions (*shout* and *show self*) that remained ambiguous. This multi-stage filtering process yielded the final, robust set of 40 actions used in the HR-ACT Database. Following this selection, we further refined these 40 animations to standardize their duration (6 s) and to ensure a consistent neutral starting and ending posture.

**Fig. 3:**
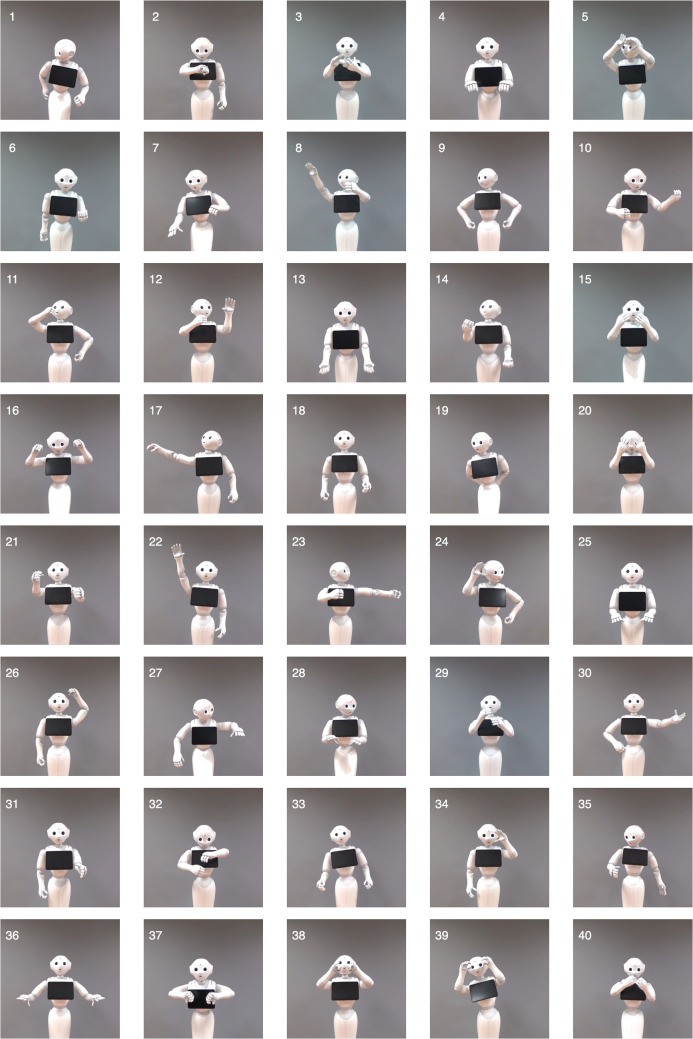
Representative frames extracted from the videos in the database for the 40 actions performed by the robot actor. The same numbering is used for these frames as for the action names and descriptions provided in Table 1

**Fig. 4:**
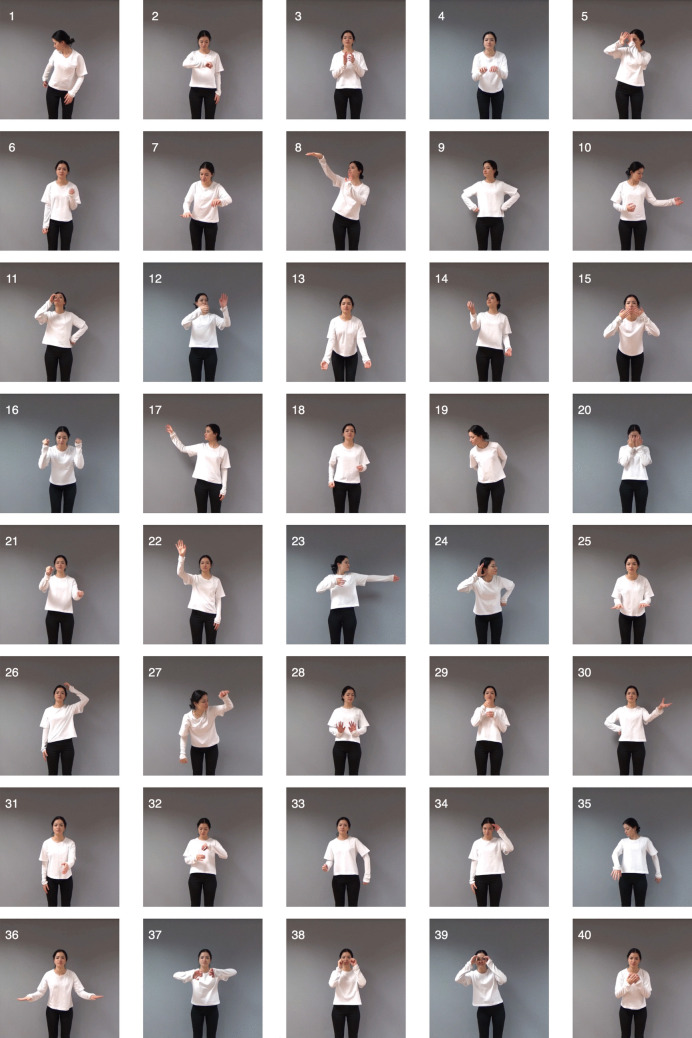
Representative frames extracted from the videos in the database for the 40 actions performed by the human actor. The same numbering is used for these frames as for the action names and descriptions provided in Table 1

Table 1 presents the final set of 40 actions selected through the pre-norming process for inclusion in the HR-ACT Database, along with their standardized behavioral descriptions. Figures 3 and 4 display representative frames extracted from the videos in the database for these 40 actions, performed by the robot and human actors, respectively. The numbering of the frames corresponds to the action names and descriptions listed in Table 1.

### Normative study

#### Video recording and post-processing

We recorded all actions using a tripod-mounted Sony HDR-CX405 camera against a dark gray curtain background, which provided optimal contrast with the predominantly white-colored actors. The recordings were conducted over two consecutive days to accommodate camera positioning adjustments required by the actors’ height differences. Using Microsoft Photos App, we created standardized 6-s clips from the raw footage. These clips were then processed using FFmpeg software (FFmpeg, 2000) to create uniform videos: muted, cropped to 1:1 aspect ratio, and adjusted to 1080x1080 resolution in .mp4 format.

Visual consistency between actors was achieved through careful wardrobe selection: the human actor wore a moderately loose white t-shirt over a matching tight blouse, which minimized skin-tone distractions while highlighting arm joints similar to the robot’s articulation points. We established precise positioning markers based on the actor’s measurements to maintain consistent spatial proportions and lighting relative to the Pepper robot’s recordings. To ensure movement accuracy, the human actor studied multiple performances of each robot action before filming, and recordings continued until her movements precisely matched the robot’s trajectory and timing.

**Table 1:** Names and descriptions of communicative and noncommunicative actions performed by human and robot actors in the HR-ACT Database

Action	Description
1. Itching	Scratches the lower back area with one hand
2. Check time	Raises arm and looks at a hypothetical watch on wrist
3. Clap	Repeatedly claps hands together in front of torso
4. Typing	Moves fingers on a hypothetical keyboard in typing motion
5. Shield	Raises both arms diagonally upward in protective gesture
6. Weight lifting	Performs alternating lifting motions with hypothetical dumbbells
7. Bounce	Repeatedly bounces a hypothetical ball with one hand
8. Throw ball	Aims and throws a hypothetical ball in basketball shooting motion
9. Disagree	Turns head side to side with hands on hips to show disagreement
10. Instrument playing	Positions hands on a hypothetical instrument and strums
11. Drinking	Brings a hypothetical cup to mouth in drinking motion
12. Wiping	Performs spraying and wiping motions with alternating hands
13. Pick up	Bends down and lifts a hypothetical box using both hands
14. Juggling	Performs juggling motion with hypothetical balls
15. Expression of love	Blows a kiss by bringing hands to mouth and extending outward
16. Get furious	Expresses anger through head shaking and rapid arm movements
17. Place manipulation	Reaches and grasps a hypothetical object, and places it elsewhere
18. Jogging	Performs jogging motion while staying in place
19. Search	Bends forward with hands behind back, turning head side to side
20. Peek a boo	Alternately covers and uncovers eyes with both hands
21. Driving	Holds and turns a hypothetical steering wheel in driving motion
22. Wave hand	Raises right arm and waves hand in greeting gesture
23. Shoot arrow	Draws and releases a hypothetical bow and arrow
24. Listening	Cups right hand behind ear and leans forward in listening pose
25. Calm down	Moves both palms downward in calming gesture
26. Confused	Scratches head while displaying confusion through head movement
27. Swimming	Performs freestyle swimming arm motions
28. Reject	Moves palms downward and head side to side in rejection gesture
29. Eating	Brings a hypothetical food item to mouth repeatedly
30. Address	Gestures with left arm and hand in directing motion
31. Hand shaking	Extends right hand forward in handshake gesture
32. Open and pour	Mimics opening a hypothetical container and pouring motion
33. Dancing	Performs rhythmic swaying movements
34. Salute	Executes salute gesture
35. Check self	Turns head to look over both shoulders alternatively
36. Stop it	Extends palms forward in stopping gesture while shaking head
37. Pull something apart	Pulls hands apart in an opening motion of a hypothetical door
38. Crying	Rubs both eyes with hands in crying gesture
39. Look through binoculars	Holds hypothetical binoculars to eyes and looks forward
40. Give me a hug	Extends arms forward with beckoning gesture, then crosses arms

We optimized several technical aspects of the recording setup for the Pepper robot. To minimize visual distractions, we programmed the robot’s tablet through Android Studio to display a black screen instead of its default interface. Due to a temporary stiffness issue causing occasional hand and arm tremors, we recorded multiple takes of each action and selected the most stable versions for the final database. Through systematic testing, we established optimal lighting conditions and camera angles, marking precise positions for both the robot and camera setup. For actions involving whole-body movements (e.g., *swimming*) or rotations (e.g., *throwing a ball*), which caused the robot to shift from its initial position, we carefully realigned Pepper to the marked position between takes.

#### Procedure

We developed parallel online surveys for robot and human actions using Qualtrics XM platform (Qualtrics, 2022). Each survey consisted of six sequential blocks: introduction, informed consent, demographics (collecting initials, birth date, gender identity, education level, and occupation), task instructions, action evaluations, and debriefing. The action evaluation block presented 40 videos for assessment, with an optional comment section at the end. To prevent duplicate participation while maintaining anonymity, we included a warning on the human condition survey’s opening screen asking previous robot condition participants to refrain from participating and cross-checked non-identifiable demographic information between conditions. The study received ethical approval from the Human Research Ethics Committee of Bilkent University.

**Table 2:** Frequencies of education levels for participants in robot and human conditions

Survey	Primary	Secondary	High	Assoc.	Bachelor’s	Master’s	PhD
	School	School	School	Degree	Degree	Degree	
Robot	2	1	46	4	110	37	11
Human	5	0	33	10	122	38	11

Using custom JavaScript in Qualtrics, we ensured each video could only be played once to maintain data integrity and informed participants of this restriction beforehand. Following each video, participants responded to three questions: 1. An open-ended action identification (“What was the action in the video you just watched? Please identify it briefly.”), 2. A confidence rating on a five-point Likert scale (1 = “not confident at all” to 5 = “very confident”), and 3. A binary classification of the action’s communicative nature (“Yes, it does” or “No, it does not” to “Does this action include communication?” question). Videos were presented in randomized order.

#### Participants

We recruited participants from diverse locations, occupations, generations, and educational backgrounds for both the robot and human condition surveys. Participation was entirely voluntary, and no monetary compensation was provided. The distribution of participants’ educational levels is detailed in Table 2.

Data collection began with the robot condition survey, which included 220 participants. One participant was excluded for providing uniform single-word responses (“action”), resulting in a final sample of 219 participants for analysis. This sample comprised 125 females and 86 males, with eight participants’ demographic information unavailable due to technical issues. Participants’ ages ranged from 19 to 70 years ($M = 37.43$, $SD = 13.38$).

The human condition survey was completed by 230 participants. After excluding eight underage participants ($<18 \text { years}$) and three randomly selected participants to match the robot condition sample size, the final analysis included 219 participants (138 females, 78 males, and three non-binary). Participants’ ages ranged from 19 to 70 years ($M = 36.90$, $SD = 13.15$).

#### Analyses of the normative data

In the normative study, we collected three types of responses for each action:

- **Naming**: Participants provided open-ended names for the actions they observed.
- **Confidence level**: Participants rated their certainty about their identifications on a scale from 1 (not confident at all) to 5 (very confident).
- **Classification**: Participants indicated whether they considered the action communicative or noncommunicative.

We conducted the analysis and scoring following the procedures outlined by Rossion and Pourtois (2004) and Zaini et al. (2013). To systematically analyze the semantic content of participants’ interpretations, we performed a frequency-based analysis on the open-ended text responses for each of the 40 robot and human actions. Our goal was to identify and quantify convergent **meaning clusters** that represent shared understandings of an action. This process involved the following steps.

##### Data inspection

 

1. **First Round**: Two volunteer research assistants, unfamiliar with the study, reviewed the naming responses. Each was initially assigned a set of 20 actions. They created new meaning clusters whenever a response appeared five or more times. For each cluster, they documented three key elements: the action’s name, the confidence level, and the classification. After completing their initial review, they exchanged the action sets so that each reviewed the remaining 20 actions initially assessed by the other, and then discussed necessary revisions together.
2. **Second Round**: Experienced members of the project team, who had participated in designing the actions and preparing the stimuli, reviewed the classifications and action cluster names from the first round. Their insights were particularly valuable in distinguishing between intended meanings and other potential clusters. The naïve research assistants and experienced team members then collaborated to determine which responses aligned with the intended meanings and which should be categorized differently.

**Fig. 5:**
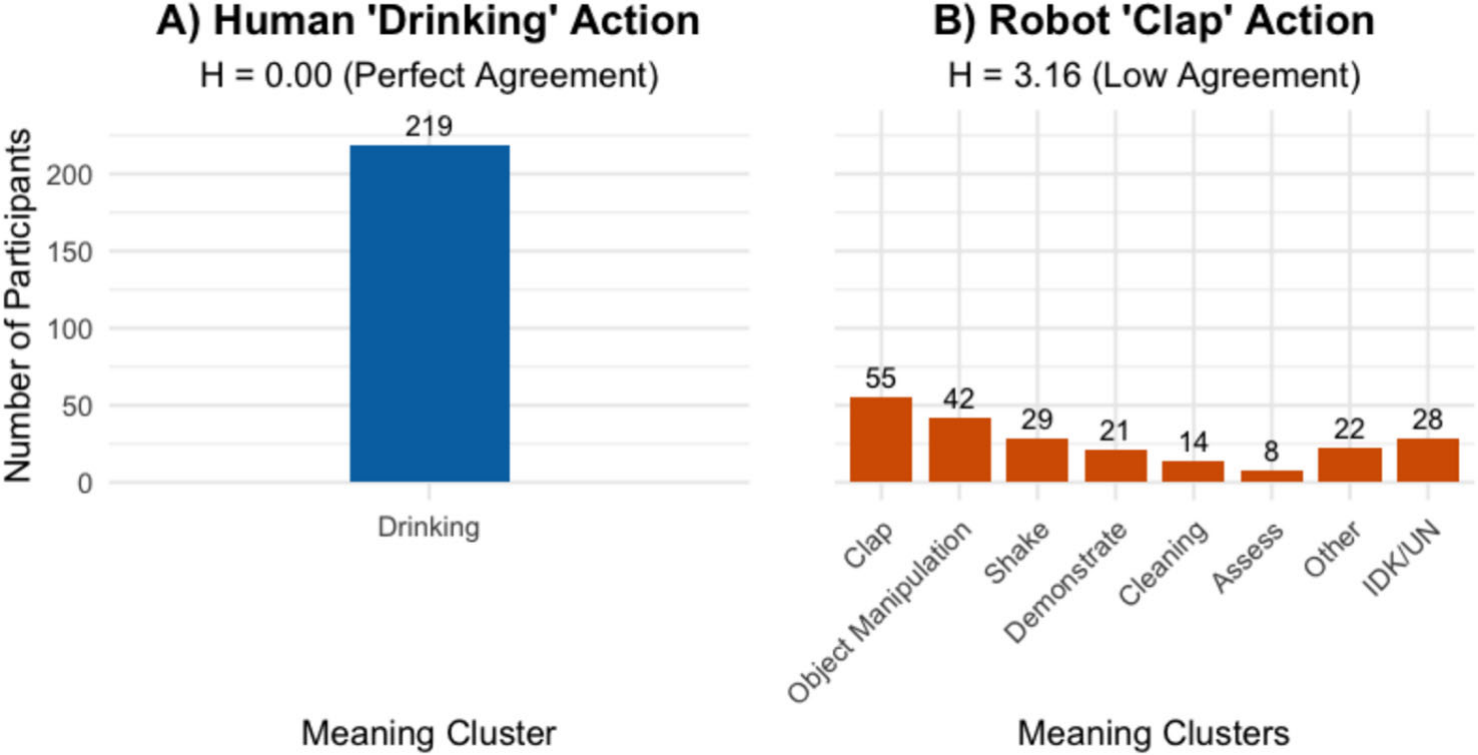
Visualization of meaning cluster distributions for actions with high versus low rater agreement. **A** The human *drinking* action elicited near-perfect agreement ($H = 0.00$), resulting in a single meaning cluster. **B** The robot *clap* action elicited low agreement ($H = 3.16$), resulting in eight distinct meaning clusters

##### Consistency check

Consistency checks were performed with the involvement of at least two members of the extended research group, with the first author consistently participating to ensure continuity in discussions. Each action and its subdivisions were thoroughly reviewed for uniformity.

##### Finalizing meaning clusters

We finalized the data structure by sorting responses for each action into four distinct conceptual groups. First, we identified the **intended meaning cluster**, which consisted of participant responses that matched the action’s designated name (e.g., *drinking*). Second, we grouped any other responses that met the frequency threshold (five or more occurrences) into one or more **alternative meaning clusters**. Third, all remaining low-frequency responses (fewer than five occurrences) were consolidated into an **‘Other’** category. Finally, responses indicating explicit confusion (e.g., “I do not know,” “I did not understand”) were grouped into a dedicated **‘IDK/IDU’** category.

For each resulting meaning cluster, we calculated its size (the number of participants who provided a response within that cluster) and its average confidence level. Furthermore, we calculated the total communicative and noncommunicative ratings for each action as a whole. All files containing the raw responses, the finalized cluster data, and the associated metrics are available in the project’s public OSF repository: https://osf.io/8vsxq/.

##### Scoring

To evaluate the level of agreement among participants for each action and cluster, we calculated the Shannon entropy $H$ (Shannon, 1948). The formula for the $H$ is presented below, where $k$ represents the number of identifications (*namings*) for each action and $p_i$ denotes the proportion of participants who provided each naming response:

$$ H = \sum _{i=1}^k p_i \log _2\left( \frac{1}{p_i}\right) $$

An $H$ of 0 signifies unanimous agreement, with all participants giving the same response, while higher $H$ indicates greater diversity in responses, suggesting less agreement among naming responses (Adlington et al., 2009; Zaini et al., 2013). $H$ is valuable as it considers the variety of meaning clusters, offering a more detailed measure than simple percentages. It is particularly useful for identifying actions that were difficult to label, or that produced a range of meanings, adding complexity to the analysis.

Our calculation of the $H$ included all meaning clusters, including ‘Other’ and ‘IDK/IDU,’ to derive more comprehensive scores. This approach ensured that even responses indicating confusion or low frequency contributed to the overall measure of agreement.

To showcase the application of our clustering and scoring methods, Fig. 5 contrasts two actions that exemplify high and low rater agreement. For instance, the human *drinking* action showed near-perfect agreement; all 219 responses converged on the intended meaning, resulting in a single cluster with an $H$ value of 0.00. In contrast, the robot *clap* action yielded high response diversity ($H$ = 3.16). While the intended meaning ‘clap’ formed the largest cluster (55 participants), several other distinct meaning clusters also emerged, including ‘object manipulation’ (42 participants) and ‘shake’ (29 participants), alongside sizable ‘IDK/IDU’ (28 participants) and ‘Other’ (22 participants) categories. This comparison demonstrates how our methodology effectively captures both clear consensus and semantic ambiguity.

**Table 3:** Identification, verification, and normative data for robot actions

Action	Intended	Comm	Ncomm	Mean	Num. of	*H*
	Class	Ratings	Ratings	Conf.	Clusters	
1. Itching	Ncomm	47	172	3.75	5	0.88
2. Check time	Ncomm	51	168	4.46	4	0.57
3. Clap	Comm	100	119	2.74	8	3.16
4. Typing	Ncomm	71	148	3.16	8	2.27
5. Shield	Ncomm	82	137	3.10	6	2.10
6. Weight lifting	Ncomm	28	191	3.58	4	1.02
7. Bounce	Ncomm	46	173	3.87	6	1.21
8. Throw ball	Ncomm	70	149	2.80	8	2.41
9. Disagree	Comm	164	55	3.11	8	2.28
10. Instrument playing	Ncomm	70	149	4.42	2	0.10
11. Drinking	Ncomm	31	188	4.27	2	0.23
12. Wiping	Ncomm	191	28	3.28	6	1.59
13. Pick up	Ncomm	31	188	3.52	4	0.87
14. Juggling	Ncomm	37	182	3.21	8	2.82
15. Expression of love	Comm	189	30	3.79	5	1.23
16. Get furious	Comm	148	71	3.48	8	2.00
17. Place manipulation	Ncomm	36	183	2.90	6	1.79
18. Jogging	Ncomm	26	193	4.12	2	0.18
19. Search	Ncomm	102	117	3.39	6	1.48
20. Peek a boo	Comm	189	30	3.56	8	2.18
21. Driving	Ncomm	28	191	4.17	3	0.64
22. Wave hand	Comm	216	3	4.28	4	0.46
23. Shoot arrow	Ncomm	29	190	4.45	3	0.40
24. Listening	Comm	175	44	3.78	4	0.98
25. Calm down	Comm	91	128	2.81	8	0.77
26. Confused	Ncomm	70	149	2.98	8	3.00
27. Swimming	Ncomm	20	199	4.15	3	0.29
28. Reject	Comm	167	52	3.48	5	1.80
29. Eating	Ncomm	50	169	3.28	6	2.06
30. Address	Comm	182	37	3.10	7	2.02
31. Hand shaking	Comm	172	47	3.85	4	1.17
32. Open and pour	Ncomm	28	191	3.28	3	0.79
33. Dancing	Ncomm	62	157	3.31	4	1.55
34. Salute	Comm	210	9	3.90	4	0.64
35. Check self	Ncomm	44	175	2.50	10	2.98
36. Stop it	Comm	172	47	3.00	9	2.25
37. Pull something apart	Ncomm	28	191	3.65	3	0.74
38. Crying	Comm	124	95	4.05	4	0.89
39. Look through binoculars	Ncomm	47	172	3.74	5	1.10
40. Give me a hug	Comm	147	72	2.67	7	2.21

*Note: Comm = Communicative; Ncomm = Noncommunicative; Conf. = Confidence; Num. = Number*

**Table 4:** Identification, verification, and normative data for human actions

Action	Intended	Comm	Ncomm	Mean	Num. of	*H*
	Class	Ratings	Ratings	Conf.	Clusters	
1. Itching	Ncomm	38	181	3.23	5	1.14
2. Check time	Ncomm	65	154	4.44	3	0.29
3. Clap	Comm	197	22	4.50	3	0.47
4. Typing	Ncomm	65	154	4.26	2	0.38
5. Shield	Ncomm	99	120	3.47	6	1.80
6. Weight lifting	Ncomm	28	191	4.14	3	0.52
7. Bounce	Ncomm	52	167	2.83	6	2.09
8. Throw ball	Ncomm	45	174	3.90	3	0.22
9. Disagree	Comm	213	6	4.28	3	0.57
10. Instrument playing	Ncomm	70	149	4.39	2	0.07
11. Drinking	Ncomm	33	186	4.33	1	0.00
12. Wiping	Ncomm	147	72	2.83	6	2.09
13. Pick up	Ncomm	59	160	3.15	5	1.20
14. Juggling	Ncomm	33	186	3.50	7	1.50
15. Expression of love	Comm	216	3	4.22	3	0.20
16. Get furious	Comm	125	94	2.80	7	2.39
17. Place manipulation	Ncomm	27	192	3.72	3	0.37
18. Jogging	Ncomm	34	185	3.96	4	0.53
19. Search	Ncomm	72	147	3.89	3	0.15
20. Peek a boo	Comm	180	39	3.21	7	2.25
21. Driving	Ncomm	40	179	4.39	2	0.18
22. Wave hand	Comm	216	3	4.07	4	0.42
23. Shoot arrow	Ncomm	27	192	4.26	3	0.52
24. Listening	Comm	185	34	4.23	3	0.24
25. Calm down	Comm	77	142	2.32	9	1.23
26. Confused	Ncomm	71	148	3.42	5	1.39
27. Swimming	Ncomm	25	194	4.54	2	0.07
28. Reject	Comm	215	4	4.27	3	0.27
29. Eating	Ncomm	51	168	2.66	6	2.39
30. Address	Comm	133	86	2.41	6	2.25
31. Hand shaking	Comm	208	11	4.17	3	0.46
32. Open and pour	Ncomm	30	189	3.71	3	0.44
33. Dancing	Ncomm	46	173	3.40	6	1.90
34. Salute	Comm	215	4	4.23	4	0.40
35. Check self	Ncomm	39	180	2.75	8	2.41
36. Stop it	Comm	188	31	3.13	8	2.05
37. Pull something apart	Ncomm	30	189	3.83	3	0.33
38. Crying	Comm	137	82	4.22	4	0.78
39. Look through binoculars	Ncomm	51	168	3.87	4	0.67
40. Give me a hug	Comm	204	15	3.02	8	2.46

*Note:* Comm = Communicative; Ncomm = Noncommunicative; Conf. = Confidence; Num. = Number

##### Results

Tables 3 and 4 present the normative data for the robot and human actions, respectively. These results include each action’s intended class (communicative or noncommunicative), the frequency with which participants identified them as communicative or noncommunicative, and mean confidence scores. The final two columns of each table display normative results, including the number of meaning clusters elicited by each action, encompassing the ‘IDK/IDU’ and ‘Other’ clusters, as well as the *H* values, which indicate the level of consensus by accounting for the diversity of responses for each meaning.

A systematic comparison of actor conditions revealed that human-performed actions were interpreted with significantly greater consensus than those performed by the robot. Human actions exhibited lower average *H* values ($M=0.98$, $SD=0.84$ vs. $M=1.43$, $SD=0.86$; Welch $t(77.95) = -2.37$, $p =.020$) and elicited fewer distinct meaning clusters ($M=4.40$, $SD=2.02$ vs. $M=5.45$, $SD=2.17$; Welch $t(77.61) = -2.24$, $p =.028$). The standardized mean differences for both *H* and clusters were of medium magnitude (Hedges’ $g = -0.53$ and $-0.50$, respectively), with nonparametric Wilcoxon tests corroborating these findings ($p=.014$ and .025, respectively). In contrast, while mean confidence was numerically higher for human than robot actors ($M=3.70$ vs. $M=3.52$), this difference was not statistically significant (Welch $t(75.51) = 1.33$, $p =.188$) and the effect size was small ($g = 0.29$).

These aggregate effects were informed by distinct patterns at the item level. For instance, certain noncommunicative actions were unambiguously identified for both the human and robot actors, as shown by their very low *H* values. These included actions like *drinking* (Human: $H=0.00$; Robot: $H=0.23$), *instrument playing* (Human: $H=0.07$; Robot: $H=0.10$), and *swimming* (Human: $H=0.07$; Robot: $H=0.29$). In contrast, other actions yielded widely different interpretations depending on the agent performing them. The action of *clapping*, for example, was clearly identified when performed by the human actor ($H=0.47$) but was highly ambiguous for the Pepper robot ($H=3.16$). Similarly, the communicative action *disagree* was well-understood when performed by the human ($H=0.57$) but generated significant confusion when performed by the robot ($H=2.28$). Conversely, some actions proved to be inherently ambiguous regardless of who performed them. The action *check self*, for instance, elicited low confidence ratings and high response diversity for both the human ($H=2.41$) and the robot ($H=2.98$), suggesting that the ambiguity in this case stems from the nature of the action itself rather than the agent’s execution.

Finally, we assessed classification accuracy by quantifying the proportion of ratings that matched each action’s intended class (communicative or noncommunicative). Mean accuracy was comparable for human and robot performers ($M=0.79$ vs. $M=0.75$, $p=.331$) and did not differ between intended communicative and noncommunicative actions ($M=0.79$ vs. $M=0.76$, $p=.564$). A two-way linear model confirmed the absence of a main effect for actor, intended class, or an interaction between them on recognition accuracy ($p\text {s} >.17$). This indicates that while human actions fostered greater consensus and fewer interpretations overall, the ability of observers to correctly classify an action as communicative versus noncommunicative was similar across action types and actors.

## Discussion

To address the growing need for standardized action stimuli across disciplines such as human–robot interaction, cognitive psychology, and neuroscience, we developed the HR-ACT Database. This resource includes communicative and noncommunicative actions performed by both a humanoid robot (Pepper) and a human actor, enabling direct comparisons between human and robot actions. The database consists of 80 videos (40 actions per agent) accompanied by normative data on action identification, confidence ratings, communicativeness, meaning clusters, and *H* values. These standardized stimuli are designed to support a wide range of research by providing controlled yet ecologically valid materials.

The HR-ACT Database offers a versatile tool for HRI researchers by enabling investigations into how humans perceive robot versus human actions. Following the approach of studies using the ABOT database (Phillips et al., 2018) to examine perceptions of robots based on their images, frames extracted from HR-ACT videos (Figs. 3 and 4) can be used as stimuli to explore how robots performing specific actions are perceived. These frames also allow direct comparisons with human actions across different action types.

In addition to static frames, the videos in the HR-ACT Database are well suited for neuroimaging or behavioral studies that require controlled stimuli. All actions are standardized in duration (6 s) and execution style, minimizing confounds related to timing or movement variability. The accompanying normative data, such as confidence scores, *H* values, and meaning cluster counts, allow researchers to tailor their stimulus selection based on specific experimental objectives.

The inclusion of raw .qanim files allows researchers using Pepper robot to implement these animations on their platforms for real-time experiments. For example, Pekçetin et al. (2024a) used HR-ACT normative data to identify representative communicative actions (e.g., *expression of love*, *salute*) and noncommunicative actions (e.g., *drinking*, *jogging*) for investigating how different action types influence perceptions of mind attribution to human and robot agents in dynamic, interactive settings (Pekçetin, 2024; Pekçetin et al., 2024b). This demonstrates how the database’s normative data can be leveraged to design controlled real-time experiments with validated stimuli.

The HR-ACT Database also supports innovative approaches by providing full sets of frames for each action, which can be combined into vignette-style tasks or cartoon-based storytelling experiments. By offering stimuli in multiple formats (images, videos, and raw animation files), the database contributes to ongoing debates about levels of naturalism in experimental psychology (Fan et al., 2021; Matusz et al., 2019) and HRI research (Li, 2015). Researchers can test identical actions across different modalities to explore how presentation format influences perception. Beyond these methodological contributions, the normative data we collected provides a valuable window into the cognitive mechanisms of human–robot action perception. The following sections will interpret these findings, connect them to the broader literature, and discuss their implications.

Before interpreting the theoretical significance of these findings, it is essential to note that our normative data were collected from a large (*N* = 438) and age-diverse (19–70 years) Turkish participant pool. This broad demographic provides a strong foundation for the results within this cultural context; at the same time, it is also important to acknowledge that the interpretation of social actions is known to be influenced by culture, from baseline attitudes toward robots to the meanings assigned to specific gestures (Bartneck et al., 2007; Nomura, 2017). We observed this directly in our data; for instance, the *itching* action i.e., “*scratching one’s back*” was also interpreted by some participants as “*warding off evil*,” a culturally specific gesture meaning. This demonstrates that while general cognitive mechanisms may explain the overall perception gap, the specific interpretations and ambiguities we report may also be shaped by our sample’s cultural background. Acknowledging this context is crucial for the proper application of our normative data and highlights the need for future cross-cultural research, which should also leverage wide age distributions, to build a more comprehensive understanding of human–robot action perception and test whether observed ambiguities extend across cultural contexts.

The contributions of the normative data presented in the HR-ACT Database are best understood when contextualized within the broader literature. While numerous studies have demonstrated a perception gap through distinct neural or behavioral responses to robot actions (Cross et al., 2012; Czeszumski et al., 2021; Thellman et al., 2022), our work provides, to our knowledge, the first systematic quantification of the resulting ambiguity. Our statistical analyses confirmed that the elevated *H* values we report for robot actions are not merely an interesting observation but a significant and robust finding, providing a direct measure of the perceptual uncertainty that was previously only inferred. This greater ambiguity aligns with prior research suggesting that humans process robot actions differently from human actions (Cross & Ramsey, 2021; Henschel et al., 2020) and may stem from a mismatch between sensory information and prior experiences, as proposed by predictive coding frameworks (Kilner et al., 2007). Our extensive lifetime exposure to human movements provides us with finely tuned predictive models, whereas our limited experience with robots may make their actions more cognitively demanding to interpret. Crucially, our item-level analysis revealed that this effect is not uniform; the human advantage in clarity is most pronounced for specific social and affective gestures (e.g., *clapping*, *disagree*), whose meanings are heavily reliant on subtle, learned cues. In this light, our database offers more than a set of stimuli; it establishes a quantitative baseline for the perception gap, enabling future studies to systematically measure the factors that might reduce it.

A critical question arising from these normative data is whether a specific $H$ value threshold exists beyond which an action’s label should not be trusted. However, our review of the relevant literature indicates that no single, theoretically driven $H$ value is commonly accepted as a standard for stimulus validation. In the absence of such a universal criterion, our approach was guided by the work of Rossion and Pourtois (2004), who presented such metrics descriptively to allow researchers to select their own stimuli from a set of complete normed stimuli based on their own experimental criteria. This contrasts with another foundational study for our work, Zaini et al. (2013), which used an author-defined threshold as an exclusionary filter, removing actions with an $H$ value greater than 1.5 to ensure high recognition agreement for those included in their stimulus bank.

A hypothetical application of this threshold to our data illustrates the rationale for our descriptive approach. This criterion would remove 18 robot actions but only 11 human actions, with ten of these actions being common to both lists. The main issue this reveals is that a uniform threshold, while filtering out some ambiguous human actions, would eliminate nearly half of the entire robot dataset. Far from being a simple methodological artifact, this difference is a core finding of our study: the ambiguity of actions, particularly those having communicative content, is significantly amplified when performed by a robotic agent. The fact that a uniform threshold would filter the two datasets so unevenly highlights the challenge of applying a single validation standard across different agent types and reinforces the value of providing complete, unfiltered normative data for future research.

The greater ambiguity observed in the robot’s actions, particularly for certain gestures, can be partly understood by considering the physical design and constraints of the Pepper robot. The development of the HR-ACT Database provided valuable insights into these factors. For instance, the robot’s friendly appearance, characterized by an open, neutral mouth often perceived as positive, might have affected interpretations of actions intended to convey negative emotions. Participants frequently interpreted furious actions as playful or childlike behavior rather than expressions of anger. Additionally, despite efforts to standardize movements between the robot and human actors, certain physical constraints of the Pepper robot necessitated modifications in action execution. For example, a tablet mounted on the robot’s torso limited the natural execution of actions requiring close contact with the torso, such as *clapping* or *hugging*, potentially contributing to the very ambiguity we measured for these actions. Finally, it is important to acknowledge that all actions in this database were presented in isolation. In real-world interactions, broader social and environmental cues would likely constrain interpretation and could enhance recognition accuracy for both agents, a factor that future research using these stimuli can systematically explore.

## Conclusion

The HR-ACT Database provides a comprehensive and versatile resource designed to support diverse research applications in human–robot interaction, cognitive psychology, and neuroscience. By offering a large set of standardized action stimuli in multiple formats (videos, image frames, and raw .qanim animation files) complemented by detailed normative data, this database addresses a critical need for controlled, replicable materials for studying action perception. The utility of the normative data, particularly the *H* value metrics for quantifying ambiguity, is demonstrated by our own analysis, which successfully quantified the significant differences in perceptual clarity between human and robotic agents. This work thus delivers not only a ready-to-use set of research materials but also a powerful methodological framework, empowering a wide range of future studies into the cognitive, cultural, and design factors that shape how we understand and interact with artificial agents.

## Data Availability

The data and associated materials of the HR-ACT Database are available on the Open Science Framework (OSF) at https://osf.io/8vsxq/. Access to the project for academic research purposes may be requested via the repository. None of the online surveys described in this study was preregistered.

## Code Availability

The code used to conduct the analyses reported in this manuscript is available on the OSF at https://osf.io/8vsxq/.

## Author note

Tuǧçe Nur Pekçetin, who was a Ph.D. candidate in Cognitive Science at Middle East Technical University during the database creation, was a postdoctoral research fellow at the Aysel Sabuncu Brain Research Center, Bilkent University during the manuscript writing, and is now a Fulbright visiting scholar in the Department of Computer Science at the University of Wisconsin-Madison. Gaye Aşkın, a master’s student in the Department of Psychology at Bilkent University at the time of the study, is now pursuing a Ph.D. in the Department of Philosophy and Cognitive Science at Lund University. Şeyda Evsen, who was a master’s student in the Department of Neuroscience at Bilkent University when the database was created, has since progressed to a Ph.D. student in the same department. Tuvana Dilan Karaduman, a recent graduate of the Department of Psychology at Bilkent University during the database creation, is now a Ph.D. student in the Department of Neuroscience at the same institution. Badel Barinal and Jana Tunç were bachelor’s students in the Department of Psychology at Middle East Technical University when the study began. Badel subsequently completed a master’s degree in the Behavioural Science Research Programme at Radboud University, and Jana is now pursuing a master’s degree in the Department of Cognitive Science at Ecole Normale Supérieure, PSL University. These authors were members of the Cognitive Computational Neuroscience Lab (PI: Burcu A. Urgen) at Bilkent University throughout the creation of the database.
